# Supplementary material for: Refined mechanism of promoter nucleosome-depleted regions resetting after replication
Source: Nucleic Acids Res. 2025 Oct 22;53(19):gkaf1025. doi: 10.1093/nar/gkaf1025 (PMC12541377; doi:10.1093/nar/gkaf1025)
Supplement: gkaf1025_Supplemental_File [file gkaf1025_supplemental_file.pdf]

Figure S1–related to Figure 1

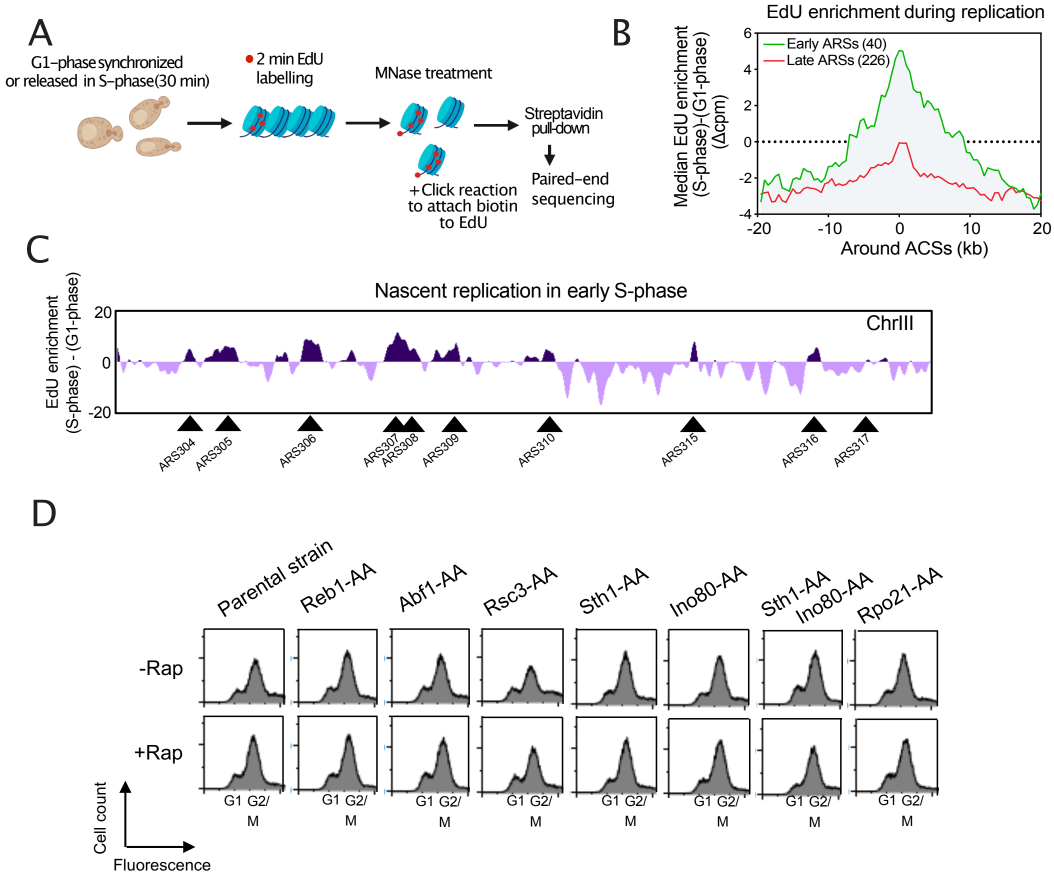

Figure S1 – related to Fig. 1:
(**A**) Schematic representation of the experimental design to define EdU labeling efficiency for purification of nascent chromatin: Cells were synchronized in G1-phase for 2.5 hr before being released in S-phase for 30min at 30°C. EdU was added in the medium for 2min during G1 arrest or at 30min after S-phase release. Cells were then crosslinked, the chromatin was extracted and treated with MNase. After a click reaction to attach biotin, newly synthesized DNA was purified with streptavidin beads. The nascent DNA was finally paired-end sequenced. (**B**) Metagene plot representing the median of the difference between S- and G1-phase cells. Replication origins were sorted as two classes: Early ARSs and Late ARSs, based on (44). (**C**) Difference between S- and G1-phase for the whole chromosome III. Early ARSs are indicated by arrows. (**D**) Flow cytometry analysis of the different unsynchronized cells treated or not with rapamycin for 30min.

Figure S2–related to Figure 1

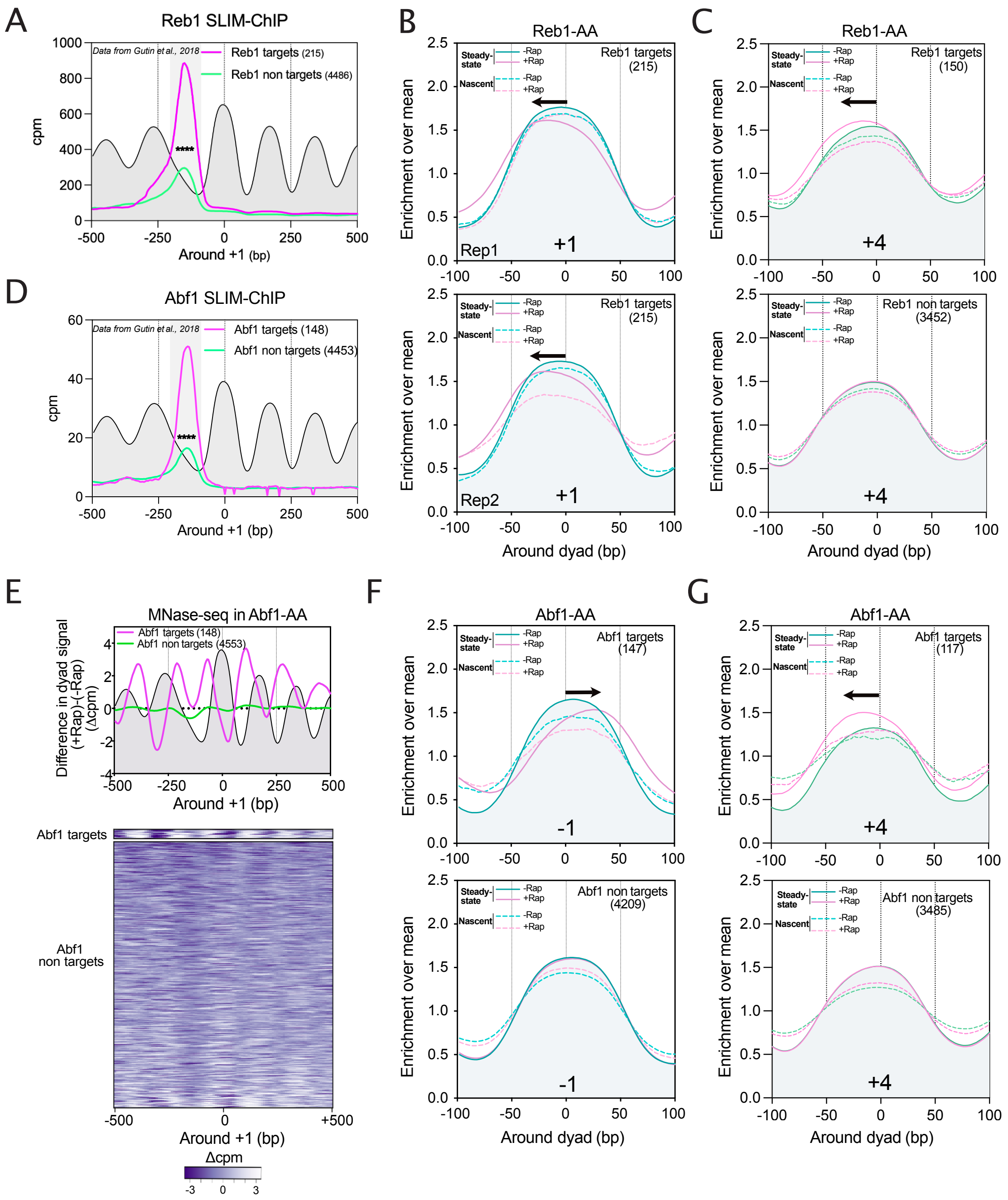

Figure S2 – related to Fig. 1:
(**A**) Metagene analysis of Reb1 enrichment at Reb1 targets and non targets. Statistics were calculated in the -200/-100bp window. (**B**) Same as Fig. 1C for Reb1 targets at +1 for each of the two replicates. (**C**) Same as Fig. 1C for Reb1 targets and non targets at +4 nucleosomes. (**D**) Same as **(A)** for Abf1. **(E)** Same as Fig. 1B for Abf1-AA. **(F)** Same as Fig. 1E at -1 nucleosome and **(G)** at +4 nucleosome.

Figure S3 –related to Figure 2

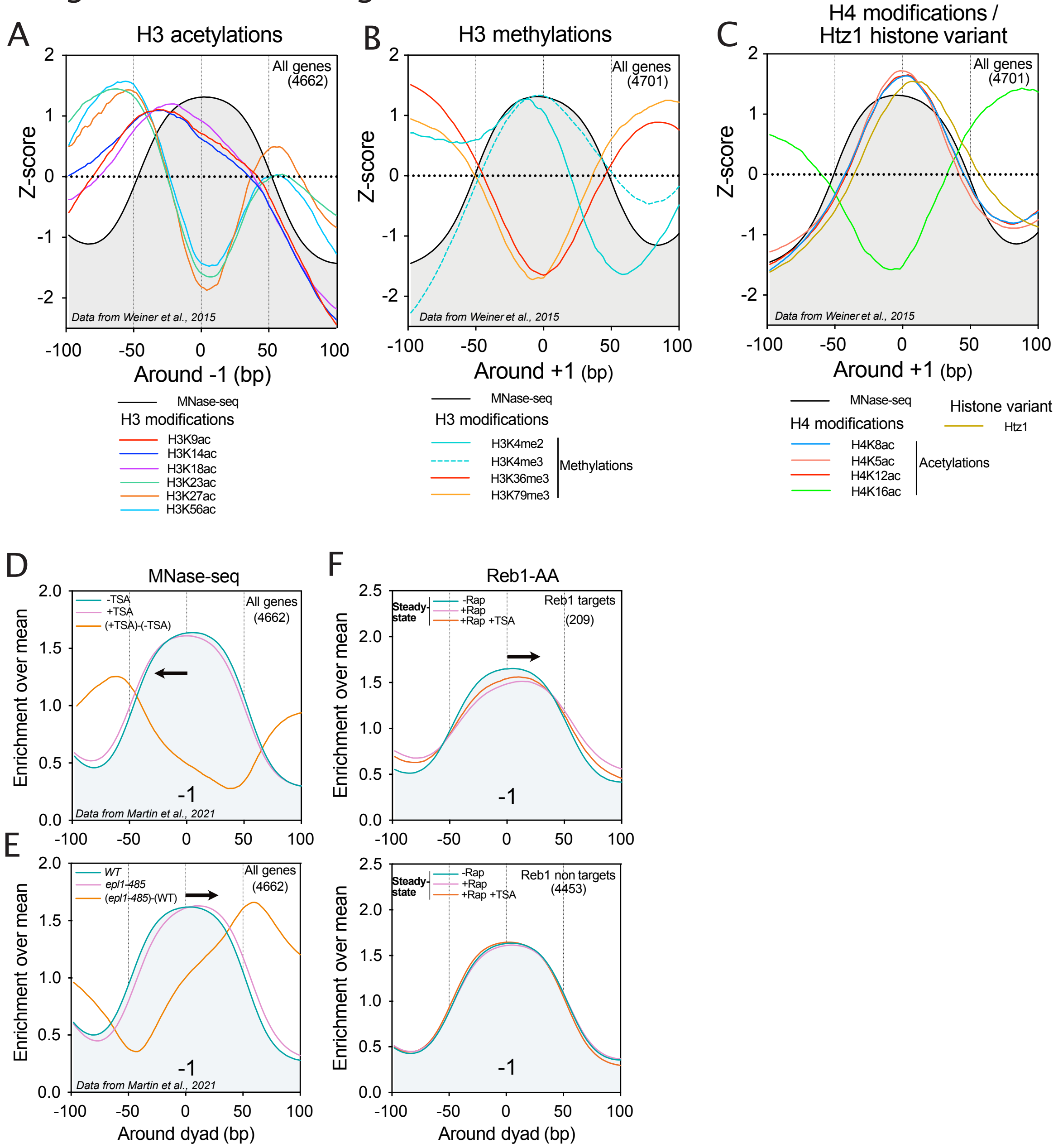

Figure S3 – related to Fig. 2:
(**A**)(**B**)(**C**) Same as Fig. 2B, respectively, for acetylation of different lysine residues on histone H3 around the -1 dyad, for methylation of different lysine residues on histone H3 around the +1 dyad and for acetylation of different lysine residues on histone H4 around the +1 dyad using data from Weiner et al., 2015. (**D**)(**E**)(**F**) Same as Fig. 2C, E and F, respectively, for the -1 nucleosome.

Figure S4 –related to Figure 3

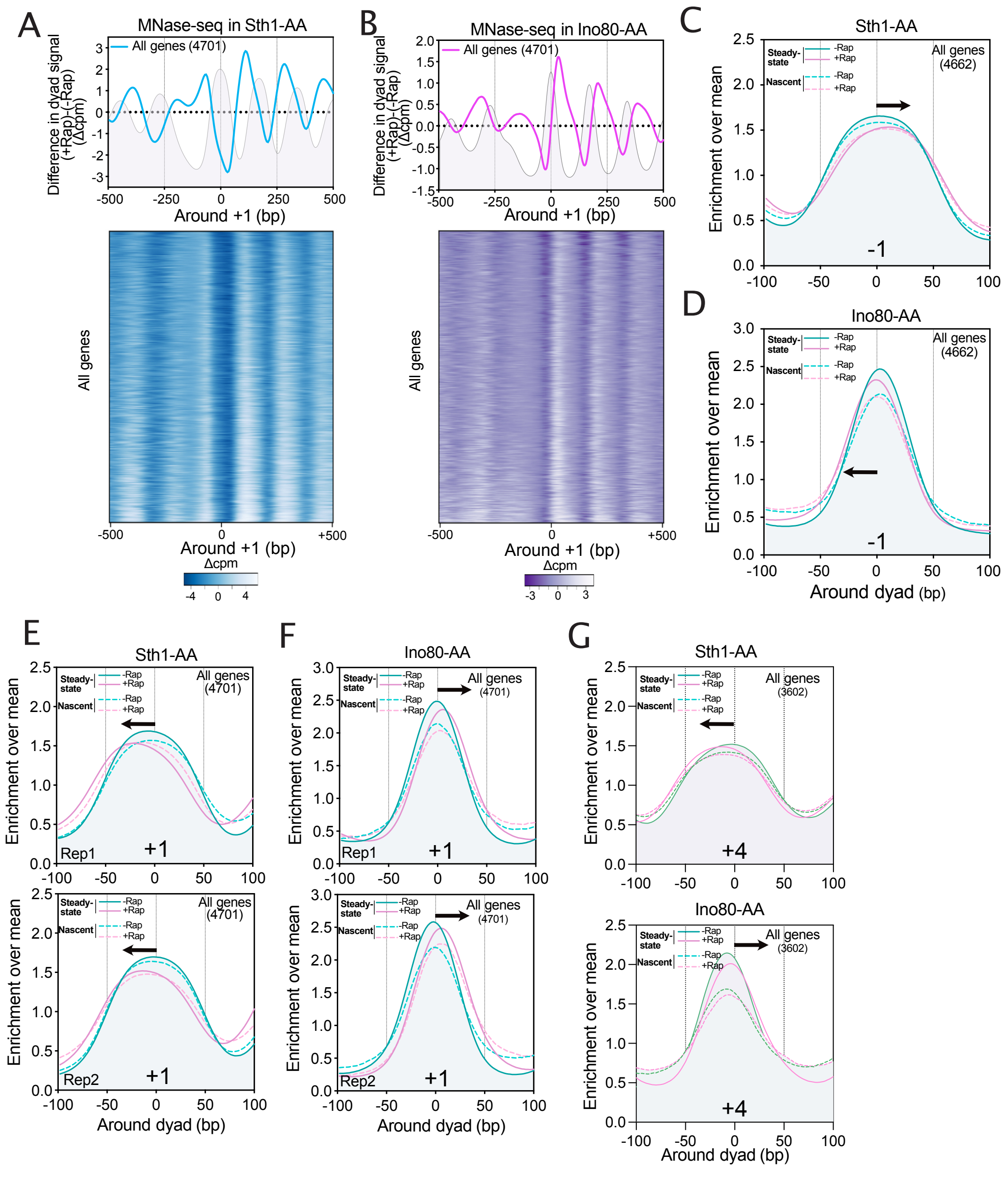

Figure S4-related to Fig. 3:
(**A**) (**B**) Average metagene plot and heatmaps of the difference in MNase-seq profiles for Sth1-AA (A) and Ino80-AA (B) strains. The grey profile represents the MNase-seq signal in the absence of Rap, in order to visualize the position of -1/+1 nucleosomes. (**C**) (**D**) Same as Fig. 3, A and C for the -1 nucleosome. (**E**) (**F**) Same as Fig. 3, A and C for each replicate. (**G**) Same as Fig. 3, A and C for +4 nucleosome.

Figure S5-related to Figure 3

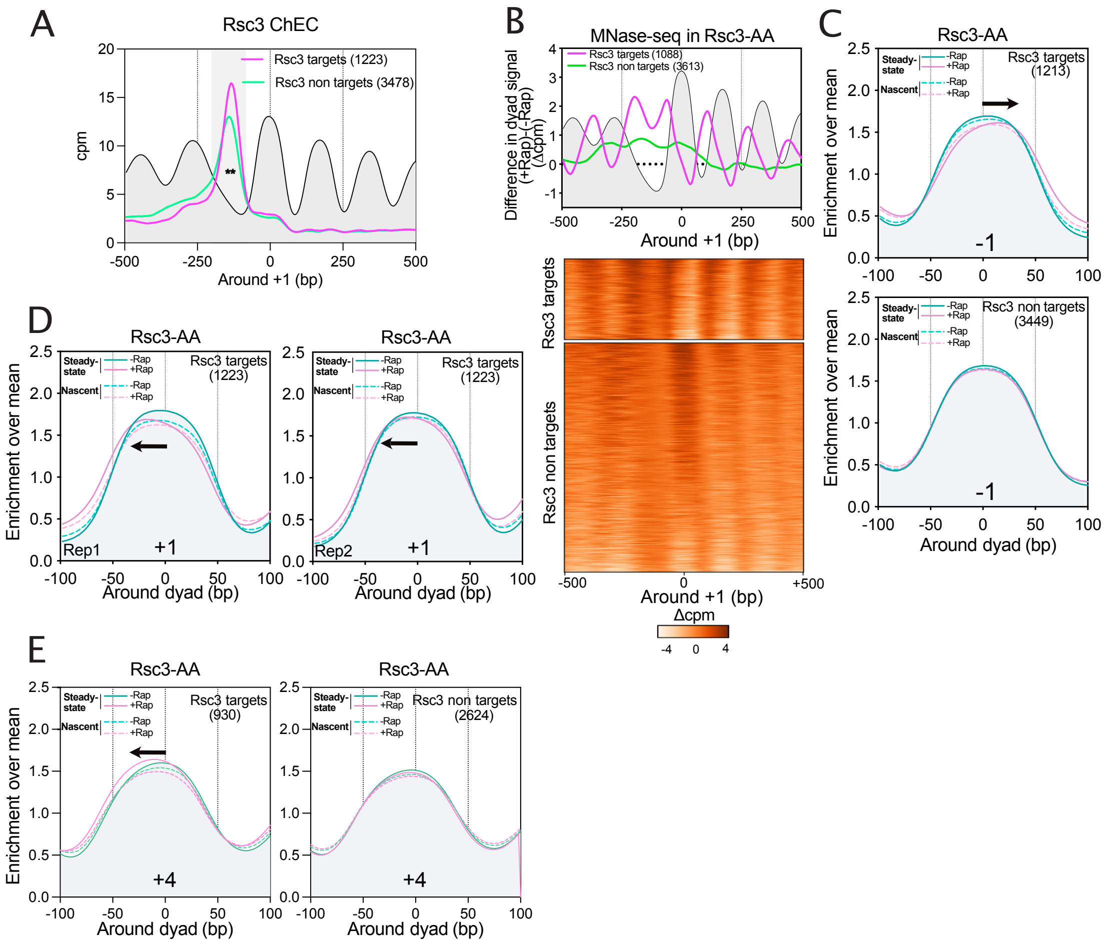

Figure S5-related to Fig. 4:
(**A**) Metagene analysis of Rsc3 enrichment at Rsc3 targets and non targets. Statistics were calculated in the -200/-100bp window. (**B**) Average metagene plot and heatmaps of the difference in MNase-seq profiles for the Rsc3-AA strain. (**C**) Same as Fig. 3E for the -1 nucleosome at Rsc3 targets and non targets. (**D**) Same as Fig. 3E for replicates at Rsc3 targets. **(E)** Same as Fig. 3E for the +4 nucleosome.

Figure S6–related to Figure 4

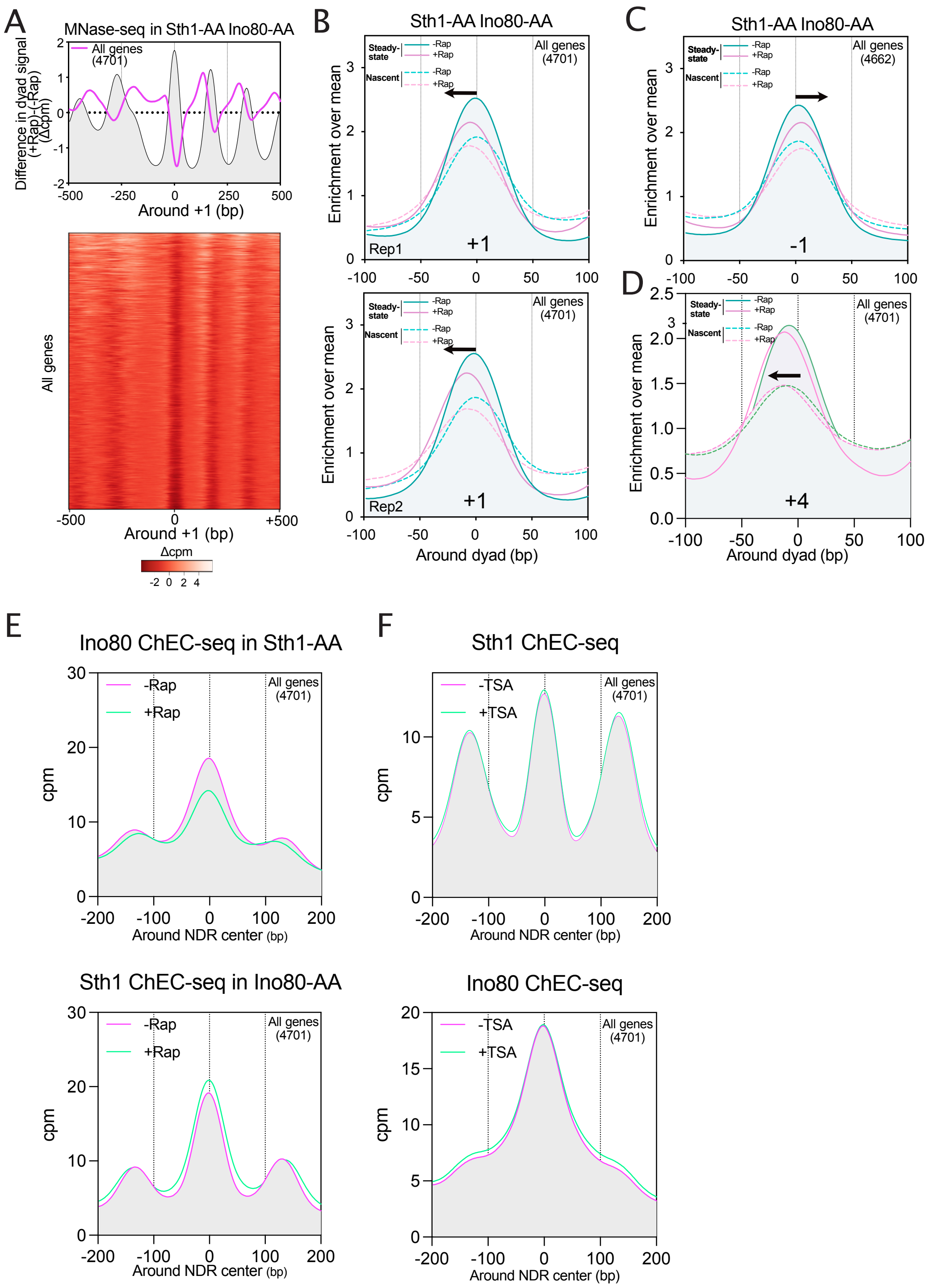

Figure S6-related to Fig. 4:
(**A**) Average metagene plot and heatmaps of the difference in MNase-seq profiles for the double Sth1-AA Ino80-AA strain. (**B**) Same as Fig. 4A for each of the two replicates at +1 nucleosome. (**C**)(**D**) Same as Fig. 4A for the -1 and +4 nucleosomes. (**E**)(**F**) Raw data of Figures 3D and E.

Figure S7–related to Figure 6

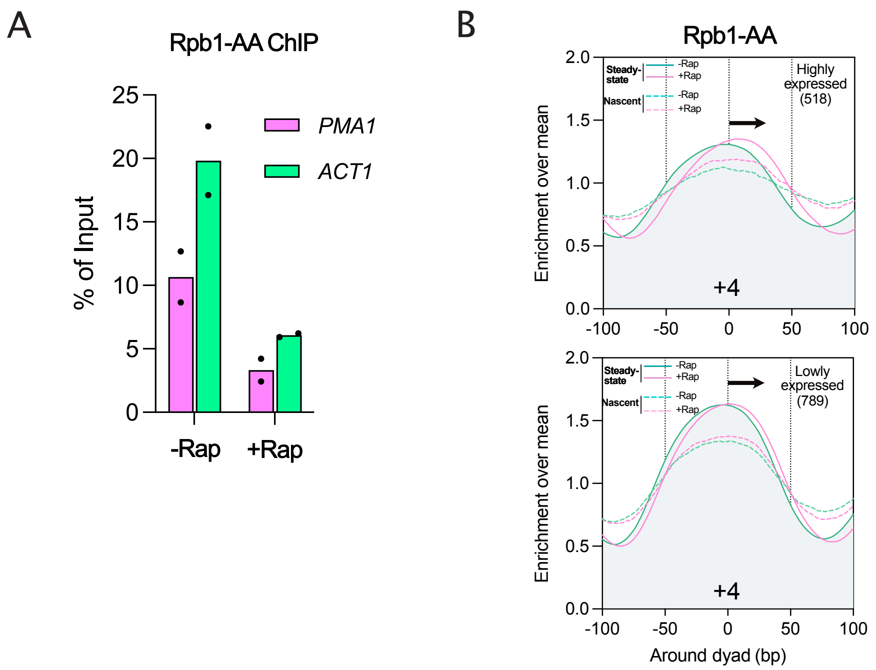

Figure S7-related to Fig. 6:
(**A**) Rpb1 ChIP at *PMA1* and *ACT1* upon 30min of rapamycin treatment in Rpb1-AA strain. **(B)** As Figure 6B with +4 nucleosome.
